# Brain edema formation correlates with perfusion deficit during the first six hours after experimental subarachnoid hemorrhage in rats

**DOI:** 10.1186/2040-7378-4-8

**Published:** 2012-07-13

**Authors:** Thomas Westermaier, Christian Stetter, Furat Raslan, Giles Hamilton Vince, Ralf-Ingo Ernestus

**Affiliations:** 1 Department of Neurosurgery, University of Würzburg, Josef-Schneider-Str. 11, Würzburg, 97080, Germany

**Keywords:** Subarachnoid hemorrhage, Cerebral blood flow, Brain ischemia, Brain edema, Animal models

## Abstract

**Background:**

Severe brain edema is observed in a number of patients suffering from subarachnoid hemorrhage (SAH). Little is known about its pathogenesis and time-course in the first hours after SAH. This study was performed to investigate the development of brain edema and its correlation with brain perfusion after experimental SAH.

**Methods:**

Male Sprague–Dawley rats, randomly assigned to one of six groups (n = 8), were subjected to SAH using the endovascular filament model or underwent a sham operation. Animals were sacrificed 15, 30, 60, 180 or 360 minutes after SAH. Intracranial pressure (ICP), mean arterial blood pressure (MABP), cerebral perfusion pressure (CPP) and bilateral local cerebral blood flow (LCBF) were continuously measured. Brain water content (BWC) was determined by the wet/dry-weight method.

**Results:**

After SAH, CPP and LCBF rapidly decreased. The decline of LCBF markedly exceeded the decline of CPP and persisted until the end of the observation period. BWC continuously increased. A significant correlation was observed between the BWC and the extent of the perfusion deficit in animals sacrificed after 180 and 360 minutes.

**Conclusions:**

The significant correlation with the perfusion deficit after SAH suggests that the development of brain edema is related to the extent of ischemia and acute vasoconstriction in the first hours after SAH.

## Background

Severe brain edema is observed in a number of patients after aneurysmal subarachnoid hemorrhage (SAH) and occurs irrespective of vasospasm-related territorial infarction. Global brain swelling is observed in 20% of SAH patients and is an independent risk factor for poor outcome [1]. However, information about the pathogenesis and the time-course of brain edema in the first hours after SAH is scarce. To date, experimental studies on the development of brain edema after SAH have covered the first 2 – 3 days after induction of hemorrhage. However, the course of brain edema in the first hours after SAH has not been investigated yet [2,3], although information about a possible treatment and its timing might be derived from this data. This study was conducted to examine the development of brain swelling and its correlation to hemodynamic and perfusion parameters in the first six hours after experimental SAH in rats.

## Materials and methods

For the experiments, 48 male Sprague–Dawley rats weighing 250–300 g were used. The animals were purchased from Harlan Winkelmann (Borchen, Germany). Animal care and experimental procedures were conducted according to the applicable governmental and institutional guidelines. All experiments were approved by the regional authorities and the district government of Bavaria, Germany.

### Anesthesia

Prior to surgical procedures, the animals had free access to food and water. The animals were anesthetized with 4% Isoflurane, orally intubated and mechanically ventilated with an air/oxygen mixture using a small-animal respirator. Throughout the experiment, pressure-controlled ventilation was adapted according to repeatedly measured arterial blood gases in order to keep those within the normal range. For surgical procedures, Isoflurane was reduced to 2.5%. After surgical preparation, 30 minutes before the induction of SAH, Isoflurane was reduced to 2% until the end of the monitoring period. Temporalis muscle and rectal probes were used to monitor the skull-base and rectal temperature throughout the experiment. A thermostatically regulated, feedback-controlled heating lamp was used to maintain temporalis muscle and rectal temperature at 37°C. The tail artery was cannulated for continuous measurement of mean arterial blood pressure (MABP), and for blood sampling. Arterial blood gases were measured 30 minutes and 5 minutes before SAH, and in hourly intervals thereafter, and before termination of the experiment and removal of the brain. Blood gases were analyzed by a Bayer RapidLab® 865 blood gas analyzer.

### Laser Doppler flowmetry and ICP measurement

LCBF was continuously monitored by a two-channel laser Doppler flowmeter (LDF) (MBF3D; Moor Instruments, Axminster, England) bilaterally through two burr holes drilled 5 mm lateral and 1 mm posterior to the bregma without injury to the dura mater. The animals were placed in a supine position with the head firmly fixed in a stereotactic frame with earbars. The rectangularly bent LDF-probes were positioned into the burr holes by a micromanipulator.

For ICP measurement, an additional burr hole was drilled over the right frontal cortex. After the animal had been brought in a supine position, an intraparenchymal Camino ICP-probe (Integra Neurosciences, Plainsboro, NJ, USA) was advanced 2 mm into the brain by a third micromanipulator.

### Induction of SAH

SAH was induced by the endovascular filament model as described by Bederson and coworkers [4]. After surgical exposure of the right cervical carotid bifurcation, temporary aneurysm clips were placed on the common and internal carotid artery. A 3–0 Prolene^©^ filament (Ethicon, Inc., Somerville, NJ) was inserted into the external carotid artery and fixed with a silk ligature. Then, the temporary clips were removed. After a recovery period of 30 minutes, the filament was advanced into the internal carotid artery (ICA). An ipsilateral decrease of LCBF after advancing the filament approximately 25 mm indicated that the tip of the filament occluded the MCA at the intracranial bifurcation of the ICA. The filament was advanced 2–3 mm further for intracranial vessel perforation, then withdrawn into the external carotid artery for reperfusion and development of SAH. SAH was indicated by a rapid bilateral decrease of LCBF and increase of ICP.

### Experimental groups

The rats were randomly assigned to one of six groups (n = 8 for each group):

1) Sham-operated animals. The filament was advanced into the internal carotid artery without perforation of the vessel and then withdrawn. The animals were sacrificed 60 minutes later.

2) Animals sacrificed 15 minutes after SAH

3) Animals sacrificed 30 minutes after SAH

4) Animals sacrificed 60 minutes after SAH

5) Animals sacrificed 180 minutes after SAH

6) Animals sacrificed 360 minutes after SAH

### Termination of the experiment and determination of brain edema

60 minutes after sham-operation or 15, 30, 60, 180 or 360 minutes after SAH, the animals were decapitated under Isoflurane-anesthesia. The brains were immediately removed, weighed on preweighed squares of glassine paper and weighed again after drying at 100°C for 24 h. The brain water content (BWC) was calculated as (wet tissue weight - dry tissue weight)/wet tissue weight)

### Correlations of LCBF and BWC

Correlations were calculated between the BWC and the ICP-, CPP- and bilateral LCBF-values for each time point and between the BWC and the area under the curve (AUC) of ICP, CPP and the ipsi- and contralateral LCBF. The AUC of the ipsi- and contralateral LCBF was calculated using GraphPad Prism 4 statistical software as the integral of the course of LCBF with “minutes after SAH” on the x-axis and “% of baseline” on the y-axis. The AUC of the ICP and CPP were calculated as the integral of the course of ICP and CPP with “minutes after SAH” on the x-axis and the absolute value (mmHg) on the y-axis.

### Statistical analysis

Statistical analysis was performed with GraphPad Prism 4 statistical software (GraphPad Software, La Jolla, CA). Blood gases and blood glucose values prior to SAH and decapitation were analyzed by a one-way ANOVA for comparison between the groups.

MABP, ICP, CPP, ipsi- and contralateral LCBF were analyzed twofold: 1. For comparison of the values between the groups, a one-way ANOVA was used and followed by a Bonferroni correction. 2. For comparison of the values within a group - in particular for comparison with the baseline value - a one-way ANOVA for repeated measures was used, followed by a Bonferroni-correction.

BWC was analyzed by a one-way ANOVA, also followed by a Bonferroni correction. Correlations were determined calculating the Pearson correlation coefficient. A p-value of <0.05 was considered significant. Results are presented as mean ± Standard Error of the Mean (SEM).

## Results

### Physiological parameters

There was no significant difference between the groups regarding pH, pCO_2_ and pO_2_ and blood glucose levels before induction of SAH, during the monitoring period or before decapitation. Values before induction of SAH and before decapitation are presented in Tables 1 and 2.

**Table 1:** Arterial blood gases and blood glucose levels prior to SAH and immediately before decapitation and removal of the brain

	pH before SAH	pH before brain removal	pCO_2_ before SAH	pCO_2_ before brain removal		
Control	7.43 ± 0.05	7.43 ± 0.06	35.4 ± 7.4	37.5 ± 6.6		
15’	7.44 ± 0.04	7.44 ± 0.04	37.6 ± 4.5	37.2 ± 4.0		
30’	7.45 ± 0.05	7.44 ± 0.05	34.9 ± 6.0	36.9 ± 6.9		
60’	7.46 ± 0.06	7.45 ± 0.05	35.9 ± 6.2	38.1 ± 6.4		
180’	7.43 ± 0.05	7.42 ± 0.09	39.2 ± 8.2	39.2 ± 5.9		
360’	7.41 ± 0.07	7.42 ± 0.05	41.6 ± 9.3	40.5 ± 5.3		

The animals were not fasted prior to the experiments. Differences between the groups were not statistically significant (mean ± SD).

**Table 2:** Arterial blood gases and blood glucose levels prior to SAH and immediately before decapitation and removal of the brain

	pO_2_ before SAH	pO_2_ before brain removal	Glucose before SAH (mg/dl)	Glucose before brain removal (mg/dl)
Control	149 ± 51	138 ± 30	356 ± 18	295 ± 12
15’	140 ± 32	143 ± 36	368 ± 16	315 ± 18
30’	153 ± 41	150 ± 45	332 ± 62	290 ± 73
60’	134 ± 14	135 ± 18	380 ± 41	326 ± 29
180’	131 ± 29	129 ± 23	356 ± 33	295 ± 17
360’	134 ± 29	128 ± 23	353 ± 12	268 ± 51

The animals were not fasted prior to the experiments. Differences between the groups were not statistically significant (mean ± SD).

### Intracranial pressure and cerebral perfusion pressure

**Figure 1:**
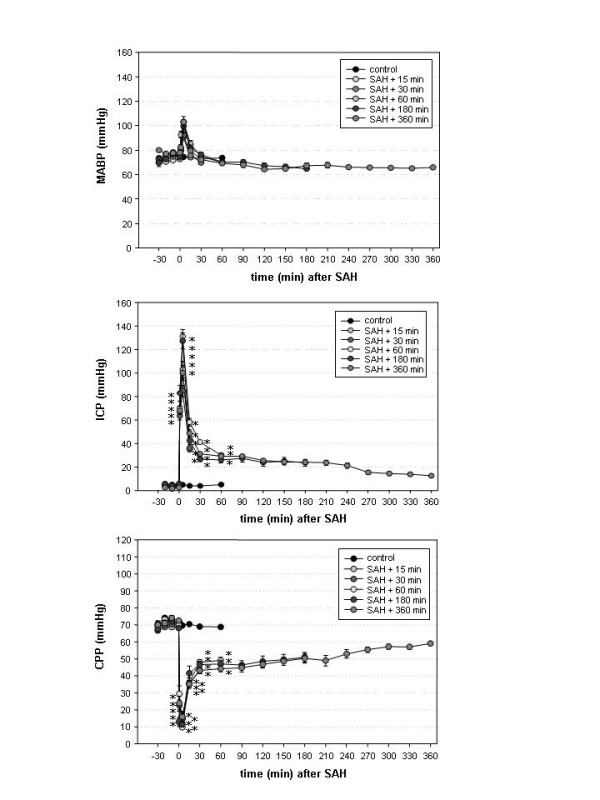
**a - c: Course of mean arterial blood pressure (MABP), intracranial pressure (ICP) and cerebral perfusion pressure (CPP) in the control group (group 1) and the SAH-groups (groups 2 – 6)**. ICP steeply increased rose after SAH in all groups and declined to only moderately elevated values within 30 minutes. MABP only moderately increased after SAH. Accordingly, CPP declined immediately after SAH, reached a peak after a few minutes but recovered until the end of the monitoring time. Results are presented as mean ± SEM (* p < 0.05 compared to the control group, one-way ANOVA).

The courses of MABP, ICP and CPP from 30 minutes before SAH until decapitation at the respective time-points are depicted in Figures 1 a - c.

Differences in MABP between the 6 groups were not significant for any time- (one-way ANOVA). Compared to baseline values, MABP was significantly elevated 5 minutes after SAH in groups 2, 4 and 6. In groups 1, 3 and 5, MABP was not significantly elevated at any time-point compared to baseline values (one-way ANOVA for repeated measures).

ICP was significantly elevated in groups 2 and 3 from induction of SAH until decapitation, and from SAH until 60 minutes thereafter in groups 4 – 6 compared to the control group. There were no significant differences between the SAH-groups (one-way ANOVA). Compared to the baseline value, ICP was significantly elevated 1 and 5 minutes after SAH in group 2, and 1, 5 and 15 minutes after SAH in groups 3 – 6 (one-way ANOVA for repeated measures).

CPP was significantly reduced versus the control group from induction of SAH until decapitation in groups 2 and 3 and from SAH until 60 minutes thereafter in groups 4 – 6. There was no significant difference between the SAH groups regarding CPP (one-way ANOVA). Compared to baseline values, CPP was reduced in groups 2 and 3 from induction of SAH until decapitation. In groups 4 and 5, the reduction of CPP was only significant 1 and 5 minutes after SAH. In group 6, CPP was not significantly reduced at any time-point (one-way ANOVA for repeated measures).

### Local cerebral blood flow (LCBF)

**Figure 2:**
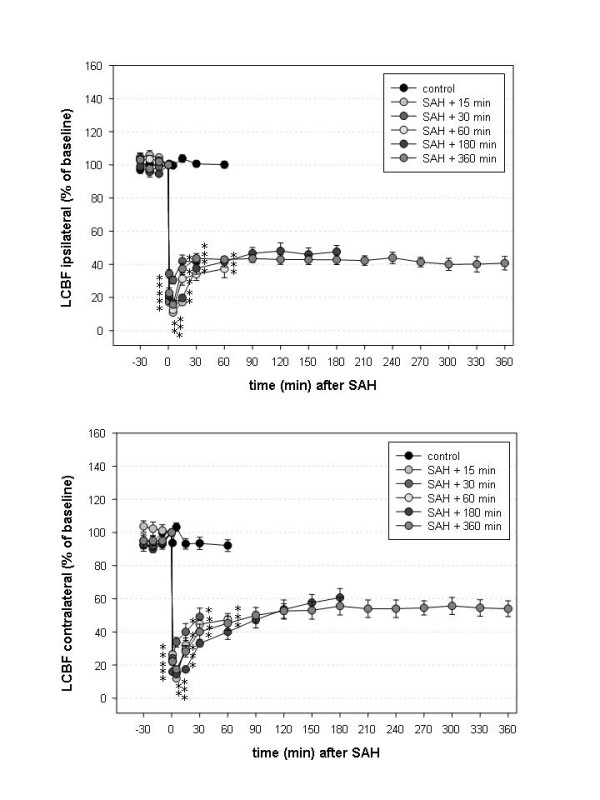
**a and b: Local cerebral blood flow (LCBF) measured over both hemispheres, ipsilateral (a) and contralateral (b) to the side of vessel perforation**. LCBF rapidly declined immediately after SAH. After a period of quick but incomplete recovery, it remained below baseline levels. LCBF recovered less than CPP suggesting an acute global vascular reaction to SAH. Results are presented as mean ± SEM (* p < 0.05 compared to the control group, one-way ANOVA).

The courses of LCBF ipsi- and contralateral to the site of vessel perforation are depicted in Figures 2 a and b. Over both hemispheres, the LCBF was significantly lower than the values of the control group from SAH until decapitation in groups 2 and 3 and from SAH until 60 minutes thereafter in groups 4 – 6. There was no significant difference between the SAH-groups at any time point on either side (one-way ANOVA). In the control group, LCBF did not significantly change throughout the monitoring period. In all SAH groups, LCBF significantly decreased compared to baseline from the onset of SAH until the animals were sacrificed (one-way ANOVA for repeated measures).

### Brain water content (BWC)

**Figure 3:**
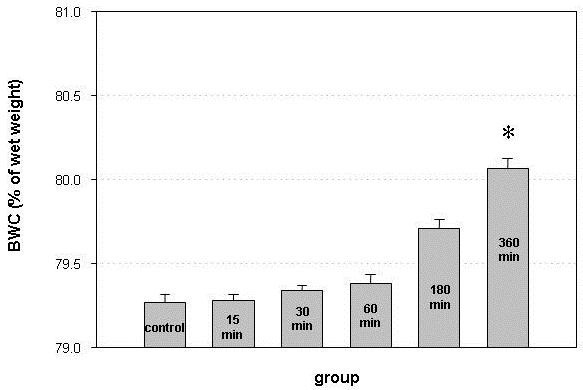
**Brain water content (BWC) in sham-operated animals (control) and in animals sacrificed 15, 30, 60, 180 and 360 minutes after induction of SAH**. BWC continuously increased in the first 6 hours after experimental SAH and was significantly higher than in sham-operated animals and animals sacrificed after 15, 30 and 60 minutes. Error bars represent SEM (* p < 0.05 compared to groups 1, 2, 3 and 4, one-way ANOVA).

The brain water content in group 1 (control group, sham-operated animals) was 79.26 ± 0.5%. In group 2 (animals sacrificed 15 minutes after SAH), it increased to 79.28 ± 0.3%. In groups 3, 4, 5 and 6 (animals sacrificed after 30, 60, 180 and 360 minutes), it increased to 79.33 ± 0.2%, 79.37 ± 0.4%, 79.71 ± 0.4% and 80.01 ± 0.5%, respectively. The differences in BWC between groups 1, 2, 3, 4 and 5 were not statistically significant (Figure 3). The increase of BWC in group 6 (animals sacrificed after 360 minutes) was statistically significant compared to group 1 (control group, p = 0.005), group 2 (p = 0.002), group 3 (p = 0.002) and group 4 (p = 0.02). The difference between group 5 and 6 was not statistically significant (p = 0.201).

### Correlations of BWC and ICP and CPP

There was no significant correlation between the BWC and any ICP- or CPP-values at any single time-point in any group. Neither was there a significant correlation between the AUC of the ICP and CPP in any group.

### Correlations of BWC and LCBF at single time-points

In animals sacrificed after 360 minutes (group 6), the extent of BWC showed a significant inverse correlation with both the ipsilateral LCBF after 180 minutes (r = −0.89, p = 0.003) and the contralateral LCBF after 180 minutes (r = −0.82, p = 0.012). The BWC did not significantly correlate to the LCBF at any earlier or later time-point in this group or to the LCBF at any time-point in the other groups.

### Correlations of BWC and the AUC of LCBF-measurements

**Figure 4:**
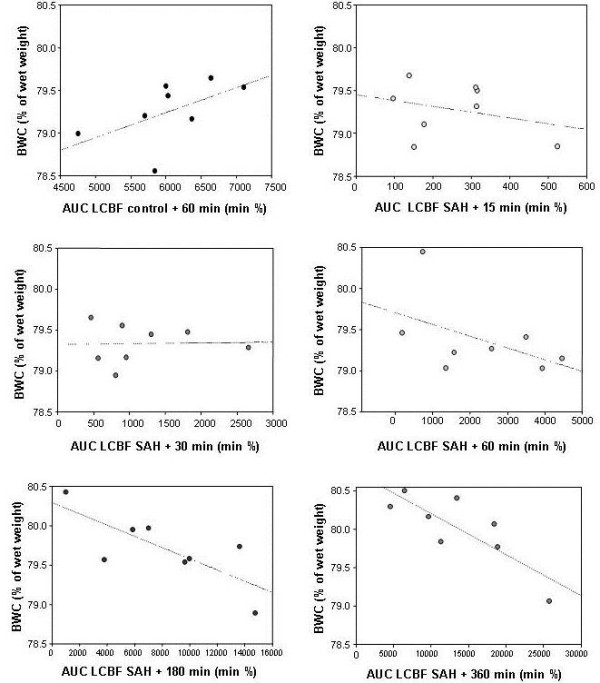
**Scatter plots representing the correlation between the AUC of ipsilateral LCBF and brain water content (BWC) in the 6 experimental groups**. Note the increasing negative correlation with increasing time after induction of SAH. Numeric values are depicted in Tables 1 and 2.

In animals sacrificed after 180 and 360 minutes (groups 5 and 6), the BWC showed a significant inverse correlation to the AUC of both ipsi- and contralateral LCBF after 180 and 360 minutes, respectively. In animals sacrificed after 15, 30 or 60 minutes, respectively, there was no significant correlation (Table 3). Correlation plots for the 6 experimental groups containing correlation curves are depicted in Figure 4.

**Table 3:** Correlations of Brain Water Content (BWC) with the Area under the Curve (AUC) of ICP-, CPP- and ipsi- and contralateral LCBF in groups 1 – 6

	**BWC vs.**	**BWC vs.**	**BWC vs.**	**BWC vs.**
	**ICP**	**CPP**	**ipsilateral LCBF**	**contralateral LCBF**
**Group 1**	0.34 (0.40)	−0.07 (0.85)	0.71 (0.06)	−0.09 (0.83)
(control)				
**Group 2**	−0.67 (0.06)	0.63 (0.09)	−0.30 (0.47)	−0.44 (0.28)
(15 minutes)				
**Group 3**	−0.58 (0.12)	0.30 (0.46)	0.03 (0.95)	0.42 (0.31)
(30 minutes)				
**Group 4**	0.07 (0.87)	0.13 (0.75)	−0.49 (0.22)	−0.52 (0.18)
(60 minutes)				
**Group 5**	−0.03 (0.93)	−0.48 (0.22)	**−0.75 (0.03)**	**−0.72 (0.04)**
(180 minutes)				
**Group 6**(360 minutes)	0.43 (0.27)	−0.01 (0.98)	**−0.82 (0.01)**	**−0.81 (0.01)**

The numbers on the left of each box resemble the correlation coefficient, the numbers on the left (in brackets) the p-value.There was no correlation of the BWC with the course of ICP- and CPP-values in any group. The BWC showed a significant inverse correlation to the AUC of both the ipsi- and contralateral LCBF-course in animals sacrificed after 180 and 360 minutes.

## Discussion

In the present study, the development of early brain edema in the first six hours after experimental SAH was investigated. To guarantee comparable conditions among the groups, all animals were kept under anesthesia and mechanically ventilated until they were sacrificed. Arterial blood gases were measured at regular intervals and kept within the normal range. We observed a continuous increase of BWC during the first six hours. There was no significant correlation between BWC and ICP or CPP. BWC and LCBF inversely correlated in the groups of animals sacrificed after 3 and 6 hours, but not in the groups of animals sacrificed after 15, 30 and 60 minutes.

The fact that the brain water content in control animals was determined after 60 minutes of monitoring may be a considered a drawback of this study. Theoretically, it might be possible that long-lasting anesthesia by itself results in progressive brain swelling. In several previous studies, however, we reproducibly observed no change of ICP with longer duration of anesthesia in sham-operated animals after 6 hours of monitoring [5,6]. At least a minor elevation of ICP should be expected if brain water content significantly increased over time. The continuous increase of BWC after SAH to levels higher than in the control group, and the fact that BWC- although not significant - was higher in animals sacrificed 60 minutes after SAH than in animals sacrificed after 60 minutes of monitoring without SAH, also indicate that the elevation of BWC is caused by SAH rather than by anesthesia of longer duration. These observations make it very unlikely that the duration of the experiment by itself has an effect on the BWC.

BWC was determined of the entire brain removed at different time points after SAH. It could be argued that pathological changes might vary between the two hemispheres, in particular because the endovascular filament model could cause endothelial damage in the large cerebral vessel trunks causing microthrombosis, a no-reflow-phenomenon and ischemia [7]. In the present experiments as well as in previous studies, however, we reproducibly observed an almost symmetric course of LCBF in both hemispheres, indicating that the observed CBF-changes affect the whole brain rather than only the side of vessel perforation [5,8]. In addition, clinical observations -show that early brain edema after SAH is global. Later in the course of the disease, at the time when secondary vasospasm occurs, focal perfusion deficits or infarctions may incite territorial swelling and mass effect [9].

To date, there is little information about the time-course and cause of early brain edema formation in the first hours after SAH. Mocco and coworkers suggested that the initial increase of ICP and decrease of CPP are igniting factors for the development of an initial cytotoxic edema. Ischemia is further maintained as cytotoxic edema keeps the ICP high and the CBF low. Persistent ischemia finally results in endothelial apoptosis, breakdown of the blood brain barrier and ultimately in vasogenic edema [10]. Our findings agree with the pathophysiological cascade suggested by Mocco et al. regarding the ischemic origin of early brain edema after SAH. However, the data indicates that different events of critical perfusion have to be distinguished after SAH. First, the sudden increase of ICP causes a decrease of CPP, sometimes up to a complete temporary cerebral circulation arrest. Within a few minutes, ICP declines and CPP gradually recovers. This pattern resembles global cerebral ischemia with gradual reperfusion. Brief periods of global cerebral ischemia have been reported to be followed by early brain edema. Warner et al. found a marked increase of BWC after 10 minutes of forebrain ischemia and 90 minutes of reperfusion in rats [11], Nakao et al. observed a significant elevation of BWC as early as 15 and 30 minutes after 15 minutes of global brain ischemia [12]. Even in three- or four-vessel-occlusion models of cerebral ischemia in rats, there is residual cerebral perfusion of up to 25% during ischemia [13] comparable to the course of CBF in the first minutes after induction of SAH in our study. Therefore, the consequences of global ischemia, like ischemic edema, which are observed in global ischemia are to be expected after SAH as well.

Second, the reduction of CBF persists while CPP already recovers. In the present study, it was less pronounced than in previous experiments with the same experimental model [5,8], however, we still observed a marked discrepancy of CPP and LCBF in all SAH-groups. This phenomenon suggests an early global perfusion deficit starting immediately after SAH and persisting until the end of the monitoring period [14,15].

The reason for this early perfusion deficit is still not clear. Delayed hypoperfusion after an episode of global ischemia may contribute to this phenomenon while the initial hyperperfusion in the first minutes after ischemia may be masked by the still elevated ICP. No-reflow phenomenon by microthrombosis or leucocyte- and thrombocyte-adhesion may additionally reduce cerebral perfusion as suggested by Hossmann and coworkers [7,16]. In addition, there may be a decisive role of early microvasospasm as postulated by Bederson et al. in his discussion of experimental findings [14] and observed by Uhl et al. during early aneurysm operations [17]. However, the latter has, to date, not been quantified in the early phase after SAH and its contribution to the early perfusion deficit is not exactly clarified. The acute global vascular reaction and perfusion deficit, may be multifactorial involving paracrine and endothelial reactions, an increased ICP and changes caused by subarachnoid blood and its degradation products.

Finally, delayed vasospasm develops in a number of patients several days after SAH and can result in infarction and focal brain swelling.

The results of the present study challenge the hypothesis of Mocco et al. in terms of to what extent the different forms of ischemia or perfusion deficit contribute to the development of early brain edema after SAH. We did observe a sharp increase of ICP and decrease of CPP immediately after SAH. Both parameters, however, did not correlate with the development of edema at any time-point. Using the same experimental model, Thal and coworkers examined the development of edema after 6, 24 and 72 hours. They observed a maximum after 24 hours [3]. Although there were certain differences in the experimental setup, the authors – similar to the results of the present study - did not observe a significant correlation of ICP, MABP or LCBF in the first minutes after SAH and BWC either. These observations suggest that SAH causes series of physiological reactions in the first minutes that occur rather uniformly and include a rapid decrease of CPP and CBF [8] and a moderate increase of BWC similar to brief periods of global ischemia. Jones et al. defined the hemodynamic thresholds for the development of cerebral infarction [18]. A decrease of CBF below one third of normal will result in cerebral infarction after several hours. In the present experiments, CPP decreased rapidly and markedly but recovered to values above 50% of baseline within a few minutes whereas LCBF remained markedly lower throughout the observation period indicating that that the sudden decrease of CPP in the first minutes after SAH may be the cause of a minor increase of the BWC in the first minutes after SAH. The correlation curves of BWC and total brain perfusion (Figure 4) show an increasingly negative correlation between 30 minutes and 6 hours after SAH suggesting that the further increase of brain edema in this period is a function of the persistent hypoperfusion after SAH. The results of Hossmann who found that the most decisive parameter for a possible recovery of brain function after prolonged global ischemia is the extent of postischemic recirculation support this hypothesis. The ability to recover function depends on the energy-dependent recovery of neurons. Similarly, the development of brain edema is caused by a loss of function of energy-dependent ion transporters, which may be reversible, if perfusion is reinstalled timely and in an adequate extent. In contrast, reperfusion after SAH is only gradual and incomplete and it is, therefore, likely that ischemic edema and other expressions of secondary brain damage increase with insufficient reperfusion.

## Conclusion

Our findings suggest that brain edema after SAH is not solely determined by a fatal few minutes immediately after hemorrhage, a time-span which eludes a therapeutic intervention as it occurs before any medical therapy can be started. It rather seems to be determined by persistent hypoperfusion. This has an immediate therapeutic implication as there might be a window for a specific treatment of early brain edema by enhancement of CBF in the first hours after SAH.

The relevance of this finding might reach beyond early brain edema. Ischemic edema is but one consequence of cerebral ischemia. A variety of other pathogenetic mechanisms like inflammatory responses, oxygen radical production or microvascular damage [19-22] are also part of the ischemic cascade and might evolve parallel to brain swelling after SAH. Therefore, timely treatment of hypoperfusion might also be effective to combat other expressions of the ischemic cascade. A pharmacological treatment of acute vasoconstriction is yet to be developed and requires further characterization of this phenomenon.

## Abbreviations

SAH, Subarachnoid hemorrhage; ICP, Intracranial pressure; MABP, Mean arterial blood pressure; CPP, Cerebral perfusion pressure; CBF, Cerebral blood flow; LCBF, Local cerebral blood flow; BWC, Brain water content; LDF, Laser-Doppler flowmetry; ICA, Internal carotid artery; AUC, Area under the curve.

## Misc

This publication was funded by the German Research Foundation (DFG) and the University of Wuerzburg in the funding programme Open Access Publishing.

## Competing interests

None of the authors has to declare financial or other competing interests to declare. The project was not subject to industrial or public funding.

## Authors’ contributions

TW – Participation in the study design, conduction of experiments, statistical analysis, drafting of the manuscript. CS – Conduction of experiments, F R – Conduction of experiments, GHV – Participation in the study design, drafting of the manuscript, RIE – Participation in the study design and coordination, critically revising the manuscript. All authors read and approved the final manuscript.
